# Strongyloidiasis: A Disease of Socioeconomic Disadvantage

**DOI:** 10.3390/ijerph13050517

**Published:** 2016-05-20

**Authors:** Meruyert Beknazarova, Harriet Whiley, Kirstin Ross

**Affiliations:** School of the Environment, Flinders University, GPO Box 2100, Adelaide 5001, Australia; harriet.whiley@flinders.edu.au (H.W.); kirstin.ross@flinders.edu.au (K.R.)

**Keywords:** *Strongyloides*, *S. stercoralis*, strongyloidiasis prevalence, global, socioeconomic status

## Abstract

Strongyloidiasis is a disease caused by soil transmitted helminths of the *Strongyloides* genus. Currently, it is predominately described as a neglected tropical disease. However, this description is misleading as it focuses on the geographical location of the disease and not the primary consideration, which is the socioeconomic conditions and poor infrastructure found within endemic regions. This classification may result in misdiagnosis and mistreatment by physicians, but more importantly, it influences how the disease is fundamentally viewed. Strongyloidiasis must be first and foremost considered as a disease of disadvantage, to ensure the correct strategies and control measures are used to prevent infection. Changing how strongyloidiasis is perceived from a geographic and clinical issue to an environmental health issue represents the first step in identifying appropriate long term control measures. This includes emphasis on environmental health controls, such as better infrastructure, sanitation and living conditions. This review explores the global prevalence of strongyloidiasis in relation to its presence in subtropical, tropical and temperate climate zones with mild and cold winters, but also explores the corresponding socioeconomic conditions of these regions. The evidence shows that strongyloidiasis is primarily determined by the socioeconomic status of the communities rather than geographic or climatic conditions. It demonstrates that strongyloidiasis should no longer be referred to as a “tropical” disease but rather a disease of disadvantage. This philosophical shift will promote the development of correct control strategies for preventing this disease of disadvantage.

## 1. Introduction

Strongyloidiasis is an underestimated disease caused by *Strongyloides stercoralis* and *Strongyloides fuelleborni*, two species of soil-transmitted helminths of the genus *Strongyloides* [1,2]. While *Strongyloides fuelleborni* is found sporadically in Africa and Papua New Guinea, *Strongyloides stercoralis* is distributed worldwide and clinically important [1]. Rhabditiform larvae of *S. stercoralis* are excreted in human feces, from where they develop into infected filariform larvae and can either repenetrate the intestinal mucosa and remain in the human organism, or distribute environmentally to new human hosts. A new host becomes infected with filariform larvae through intact skin penetration [3]. Strongyloidiasis can cause gastrointestinal symptoms, including abdominal pain, diarrhea, nausea and vomiting, skin problems including pruritus and dermatitis or respiratory symptoms such as cough, asthma and dyspnea [4,5,6]. Hyperinfection or disseminated strongyloidiasis can affect several organs, leading to fatal outcomes [1,7]. Chronic asymptomatic strongyloidiasis is another significant concern, as when coupled with immunosuppressive treatment, it has potential to develop into disseminated infection [8].

Currently, strongyloidiasis is predominately described as a neglected tropical disease, found in tropical and subtropical areas (Southeast Asia, Africa, Central and South America) [9,10,11,12,13,14]. Whilst these papers do not often include clear climate-area classifications, it seems inappropriate that the primary disease descriptor focuses on geographic and climate conditions. Recent studies have included countries of the temperate zones in the endemic areas for strongyloidiasis [15,16,17,18,19]. There are also cases of strongyloidiasis in some parts of the same climatic zone but not in others [20,21,22,23]. This indicates that climatic conditions are not the primary factors determining the disease presence. Few studies mentioned low sanitation and socioeconomic status of communities as a risk factor for strongyloidiasis, and those that did not examine socioeconomic and sanitation conditions in any depth [21,24].

This review explores the global prevalence of strongyloidiasis in relation to its presence in subtropical, tropical and temperate climate zones with mild and cold winters, but also explores the corresponding socioeconomic conditions of these regions. The review demonstrates that classifying the disease as “tropical” is misleading and runs the risk that physicians in other countries may not recognize this pathogen, resulting in misdiagnosis or mistreatment of the disease [15,25], but most importantly it influences how the disease is fundamentally viewed. Correct classification and perception of the strongyloidiasis is crucial, as it determines what strategies and control measures are used to prevent the infection. Considering the disease as an environmental health issue than a clinical issue based on geography would provoke a shift from drug administration to environmental health controls. Clinical treatment of strongyloidiasis will not always be effective [13,26]. Anthelminthic drugs do not prevent reinfection, and can also cause adverse health effects [27,28]. Additionally, resistance to ivermectin (the primary drug used to treat strongyloidiasis) has already been found in *Strongyloides* spp. infecting ruminants [29], suggesting that resistance to ivermectin in *S. stercoralis* is likely in the future. Therefore, environmental health interventions represent a safer and more effective way of infection treatment. It was more than twenty years ago that Grove [30] noted that controlling *Strongyloides* in the environment is the most effective way to reduce infection. He pointed out that installation of adequate waste disposal systems was the most effective method to control the nematode [30], although this has not become the primary approach to addressing the disease. A major step towards reducing *Strongyloides* infection is to change the global perception of strongyloidiasis as a neglected tropical disease to recognition that it is primarily a disease of disadvantage and poor sanitation.

The aim of the current review was to assess the global prevalence of *S. stercoralis* to determine prevalence in geographic locations or climate zones, and compare these with socioeconomic status and poor infrastructure of the communities. The review demonstrates that strongyloidiasis should no longer be referred to as a “tropical” disease but rather a disease of disadvantage. This philosophical shift will promote the development of correct control strategies for preventing the disease.

Studies that collectively demonstrate the global distribution of *S. stercoralis* are presented in Table 1. Studies were collated using the Google Scholar and PubMed journal databases and the key words *Strongyloides*, *S. stercoralis*, strongyloidiasis, global, socioeconomic, status. Only studies from 1990–2016, written in English or Russian, with *S. stercoralis* prevalence percentage and details on population studied were included in the review. Reference lists of the collected studies were also examined and relevant articles have been reviewed.

## 2. Global Prevalence of Strongyloidiasis and Climate Classification

Table 1 summarizes the information available on infection prevalence, population studied, country as the most likely infective source, climate and socioeconomic status of the country, type of the infection detection, presence of symptoms and the study reference details. The table indicates that all cases of strongyloidiasis occur in the following communities: poor communities, former war veterans, immigrants and travelers, immunocompromised populations, or groups occupationally exposed to soil.

**Figure 1:**
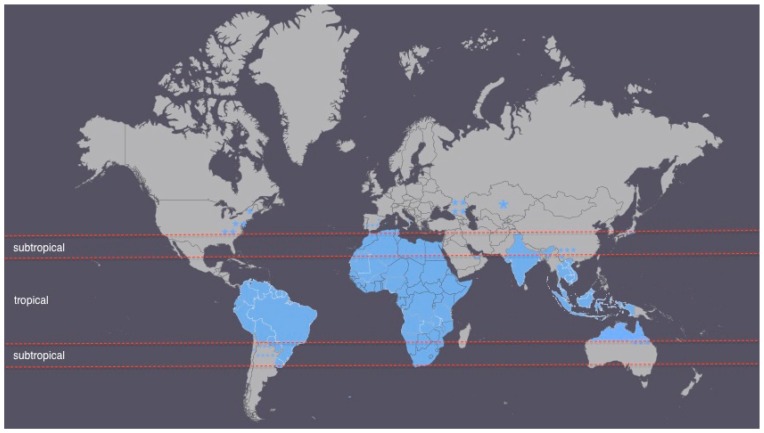
Countries with *Strongyloides stercoralis* cases (colored blue or marked as a “star” sign) on a world map divided into tropical and subtropical zones.

The climate classification used in this review is the Koppen climate classification system, which divides the world’s climate into six major climate groups each containing several subgroups [57]. Using the complete range of Koppen climate categories, about 80% of all the world areas falls into either tropical or subtropical zones [57]. This justifies the classification of strongyloidiasis as a “tropical” or “subtropical” disease, but lacks any meaning or association. Based on the major Koppen climate categories, the infection is still prevalent in other climate zones apart from tropical or subtropical ones (Figure 1). Certain areas of countries with *Strongyloides stercoralis* cases are shown as a “star” sign on the map.

Figure 1 presents a world map divided into tropical and subtropical zones with the strongyloidiasis case countries/areas colored in blue. It can be seen that strongyloidiasis is highly prevalent in subtropical and tropical regions representing mostly developing countries with low socioeconomic status. Cases outside the tropical or subtropical areas correspond to more economically developed countries, but socioeconomically depressed communities (e.g., the Appalachia region population in the U.S., former USSR countries). This emphasizes that socioeconomic factors are more important than climatic conditions in defining the disease. The remaining cases presented are in risk groups of developed economy countries such as former war veterans, refugees, immigrants and travelers, immunosuppressed people or current or ex-farmers and their families, also identified by Schär *et al.* [24].

## 3. Countries of Strongyloidiasis Prevalence and Socioeconomic Status

### 3.1. Socioeconomic Status of the Strongyloidiasis Case Communities in Subtropical and Tropical Zones (Hyperendemic)

The socioeconomic status of the countries are presented in Table 1, based on their economy status and the income using World Bank data and the United Nations “*World’s Economic Situation and Prospects 2016*”report [58,59]. It is globally accepted that an area with *S. stercoralis* prevalence of more than 5% is considered hyperendemic [60]. From Table 1 it can be seen that almost all the reported countries are shown to be hyperendemic for strongyloidiasis, with exception of the Appalachia region in the U.S., Okinawa in Japan and North Caucasus in the former USSR. The reported endemic areas for strongyloidiasis (Southeast Asia, Africa, Central and South America) are mostly countries with developing economies, as can be seen in Table 1. Socioeconomic inequalities result in poor sanitation and hygienic conditions, which act as a triggering factor for the pathogen infection [17]. The lifecycle of *S. stercoralis* and a mode of infection transmission justifies the notion that improper sanitation conditions are risk factors for infection [3]. Increased urbanization processes happening in such countries cause inappropriate living conditions for the population such as 5–6 people living in one room and the use of one cubicle shower and a toilet [32]. It has been frequently shown that low socioeconomic status communities present higher mortality and morbidity rates compared to higher socioeconomic class population [61,62].

### 3.2. Socioeconomic Status of the Strongyloidiasis Case Communities in Temperate Zones

Apart from high prevalence strongyloidiasis cases detected in most of the subtropical and tropical countries in the world, cases with strongyloidiasis prevalence were also shown in some continental climate regions (Appalachia, North Caucasus, Kazakhstan). Although the study conducted in the North Caucasus does not meet the current review’s criterion for the year of publication of papers, it is still included as not many studies from that area are available. North Caucasus has a continental climate and the study findings highlight that strongyloidiasis is not dependent only on climatic conditions [55].

While moist and warm soil, enriched with nutrients are favourable conditions for the survival of free-living *S. stercoralis* larvae with further potential to infect a human host, the factors influencing direct or indirect development of infective filariform larvae (L3) are poorly understood [1,60]. Previous reports have indicated that larvae cannot survive temperatures below 8 °C or above 40 °C [63]. However, studies have demonstrated *S. stercoralis* larvae surviving at lower temperatures infecting a human [55]. Considering the parthenogenesis and autoinfection features of this nematode, the likelihood of the larvae remaining and reproducing within the host is high. In conditions of inadequate sanitary and hygiene environment there is then a high risk of rhabditiform larvae excreted in stools passing to other human hosts.

As seen in Table 1, these regions belong to countries or a country with transitional or developed economies with the strongyloidiasis cases identified only in disadvantaged communities [21,22,55]. For example, rural Appalachia regions in Kentucky, West Virginia, Georgia and Tennessee in the United States are identified as areas with high infection prevalence among low socioeconomic status populations [21,64]. The *Strongyloides* infection case reported in Kazakhstan children were adopted children from orphanages, who probably were exposed to poor sanitary environments [22]. The study in the North Caucasus reported different levels of strongyloidiasis prevalence (0.1%–1.4%) in different areas with different temperatures (the lowest being 4 °C). Poor sanitary conditions were however reported in almost all the communities studied [55]. These single *Strongyloides* infection cases occurred in areas of continental climate, where the precipitation level is low and temperatures go below zero, demonstrating that strongyloidiasis is not primarily influenced by climate conditions but rather sanitary and hygiene factors.

Australia is known to have tropical and subtropical climates, however, strongyloidiasis there is frequently found among indigenous communities and not the general population [20,23]. Indigenous communities (Aborigines and Torres Strait Islanders) are identified as of a low socioeconomic status populations and are generally reported to live in poorer housing, sanitary and infrastructure conditions, which results in numerous worse health outcomes compared with non-indigenous Australians [65].

### 3.3. Clinical Treatment of Strongyloidiasis and Infrastructure, Housing, and Environmental Health 

Currently, anthelminthic drugs (albendazole, mebendazole, and ivermectin) and nemiticides are used to treat the strongyloidiasis in humans [66]. Treatment of soil-transmitted helminthiasis is difficult due to the development of resistance and facile reinfection from the environment. Among soil-transmitted helminth infections, strongyloidiasis is the most challenging to treat and clinically important because of a parasite’s rhabditiform larvae unique ability of autoinfection [2,13,26,32]. Moreover, parthenogenesis allows for a single female parasite remaining in a host to reproduce reinfecting that person [1]. The drug treatment efficacy depends on number of factors including an individual’s immune system status, co-infection with HTLV-1, history of drug use, and bowel ileus [51,67,68,69]. Furthermore, monitoring treatment efficacy has some difficulties associated with the low sensitivity of fecal examination [13]. Additionally, the drugs can cause adverse effects, including liver disfunction, gastrointestinal symptoms (nausea, vomiting, loose stool, abdominal distension or pain), chest tightness or pain, itching, fever, cough and wheezing, dizziness, and neurological effects [27,28,70,71].

New anthelminthic drugs and nematicides have to be frequently introduced to the market due to quick resistance development in nematodes and great toxicity they produce to humans [26]. Resistance in nematodes to different drugs has been studied and demonstrated frequently in the veterinary field in the last decades [72,73,74]. This suggests that human-infecting nematodes are also likely, at some stage in the future, to become resistant to the available drugs. Indeed, studies on some drugs used against human nematodes have already reported low drug treatment efficacy, calling for great attention and warnings of possible resistance development [75,76]. Although it is more difficult to study and confirm anthelminthic resistance in human parasites due to number of factors, the potential for resistance is mostly overlooked and should be more carefully examined in drug treatment application [77].

It is well established that sanitary conditions, including housing and infrastructure, play the most vital role in determining health outcomes [78,79]. Overcrowding, poor ventilation, bad living conditions and inadequate sewerage systems create higher risks for infectious and parasitic diseases such as skin infections, respiratory infections and diarrheal diseases [80]. Thus, environmental health approaches such as ensuring better infrastructure and sanitation should be the primary approach to controlling *Strongyloides* in the environment. Only this approach will provide the most effective way of infection reduction.

Strongyloidiasis has been also reported in certain groups such as former war veterans, refugees, immigrants and travelers, immunocompromised people and people occupationally exposed to soil (Table 1). Poor sanitary and hygiene living conditions are common during times of war, which could explain cases of strongyloidiasis in former war veterans [16,40]. The Okinawa Prefecture area of Japan was reported to have a high prevalence of *S. stercoralis* infections during World War II, which decreased to about 0.5%–1.5% after the war years. This was associated with improved sanitary conditions and systematic monitoring for parasitic diseases after the war [81].

Studies of refugees and immigrants with high *S. stercoralis* infection prevalence have demonstrated an association with inadequate sanitary and hygienic conditions in their home countries, including lack of an access to shower and toilet facilities [4,8,19,31,33,36,38,41,50].

Individual health condition (immunosuppressed or immunocompromised status) is another risk factor influencing the disease [5]. *S. stercoralis* is especially life-threatening to immunocompromised people due to possible development of the disseminated disease form [5,8] which approaches a 90% mortality rate [5,82]. The study by Zaha *et al.* [81] demonstrated that there was a high prevalence of *S. stercoralis* among Human T-Lymphotropic Virus type I (HTLV-1) positive patients (17.5%) compared to HTLV-1 negative patients (6.7%). Schar *et al.* [24] found an association between strongyloidiasis and HIV infection (OR: 2.17 BCI: 1.18–4.01) and alcoholism (OR: 6.69; BCI: 1.47–33.8). HTLV-1 and HIV infections and alcoholism have been associated with poverty [61,83,84,85].

High prevalence of strongyloidiasis in subtropical South China has been reported by Wang *et al.* [86]. While the cases reported are within subtropical areas, they are mostly associated with the farming lifestyle in those regions and/or poor hygiene practices. The infection rates in these areas are as high as 11%–14% [86]. The studies’ findings are not included in Table 1, as the original papers are only available in Chinese. Similarly, studies in France and Spain [37,42,87] reported strongyloidiasis cases in local current or ex-farmworkers and their family members who have never travelled to endemic areas. While there is no available information on the income of the studied population, ingestion of non-potable water and possible infection transmission to family members due to unhygienic behavior is reported in one of the studies [42]. This might indicate either inappropriate living conditions due to the depressed socioeconomic status in the area or population unawareness of proper hygienic and sanitary standards. On the other hand, in another study by Roman-Sanchez *et al.* [37] the assessed area (Gandia, Valencia), is reported to have the highest *per capita* income compared to other European Union regions, adequate hygiene-sanitary conditions and high prevalence of the strongyloidiasis. Whether the use of a more sensitive detection method, the agar-plate culture technique, compared to other studies impacted on this result cannot be known until several studies using the same detection tests are conducted. It can, however, be concluded that occupation is likely to contribute to acquiring the infection in this case.

Currently, it is estimated that between 30–100 million people are infected by *Strongyloides* worldwide [2,88]. There is however a general consensus amongst the scientific community that the prevalence is underestimated due to inadequate diagnostic techniques [88], and the lack of sensitivity in tests for *S. stercoralis* and the similarity of its symptoms to other diseases result in great underestimation of the infection and 300 million people infected globally is probably a more accurate estimate [47,89]. Misclassification of the disease may also be contributing to the underestimation of its prevalence. Diagnostic test methods are presented in Table 1 for completeness.

## 4. Conclusions

It is well established that strongyloidiasis is mainly restricted to tropical and subtropical areas throughout the world. However, within these regions, exposure to infection with the helminth is strongly associated with poor sanitary and living conditions. Thus, immigrants, refugees, travelers, war veterans, immunocompromised and occupationally soil-exposed groups—and their family members—are at especially high risk of strongyloidiasis. This review emphasizes that strongyloidiasis is a disease of disadvantage, and suggests that control measures to prevent the infection should focus as much, or more, on changing the environmental conditions that increase overall risks of the disease, as on the medical treatment of infected persons, especially since the latter is ineffective in preventing reinfection and has the potential for the development of drug resistance.

## Figures and Tables

**Table 1:** Global *Strongyloides stercoralis* prevalence distribution.

NO.	Most Likely Infective Source	Climate Classification	Population Studied	SES	*S. stercoralis* Prevalence (%)	Type of Detection	Symptoms Diagnosed	Comments/Details	Reference
1.	East Africa	Group A, C, B *	Immigrants (≥16) lived in the refugee camps, Melbourne community health center and clinic patients	Developing economy 1 **	11% (14/124)	Serology	Fever (34%), Stomach pain (30%), weight loss (25%), and diarrhea (13%)	Arrived to Australia, Melbourne between 1997–2000	[4]
2.	Cambodia	Group A	Immigrants and refugees (≥15), Melbourne community health center and clinic patients	Developing economy, 1	42% (97/230)	Serology	Not reported	Arrived to Australia, Melbourne between 1974–2002	[4]
3.	Laos	Group A	Immigrants (≥18)	N/a	24% (22/93)	Serology	75% (60/80) had previously worms, not known symptoms	Arrived to Australia, Melbourne between 1980–1989	[31]
4.	Brazil (North, Northeast, Midwest, Southeast, South)	Group A, C	General population	Developing economy, 3	5.5% 21.7% 29.2%	Stool examination Serology (IFAT) Serology (ELISA)	Not reported	Study conducted from 1990 to 2009	[32]
5.	Mexico, Honduras, Ethiopia, El Salvador, Zambia, Argentina, Congo, Cuba, Grenada, Guatemala, India, Kenya, Niger, Tanzania, Vietnam	Group A, C, B	HIV-positive immigrants (≥17)	Developing economy, 1,2,3	26% (33/128)	Serology	Weight loss (53%), diarrhea (48%), fatigue (42%) and abdominal pain (36%).		[5]
6.	Africa, Central/South America, Thailand, India, UAE	Group A, B, C	HIV-positive immigrants (≥18), Italian hospital patients	Developing economy, 1,2,3,4	11% (15/138)	Serology	Skin problems (16.7%), gastrointestinal symptoms (15%) respiratory problems (14%)	Study conducted from 2000 to 2009	[6]
7.	Sub-Saharan Africa	Group A, B, C	Immigrants, Royal Melbourne Hospital, Infectious disease clinic patients	Developing economy, 1	1.4% (2/145) 17.9% (32/179)	Stool examination Serology	Not reported	Study conducted from 2003 to 2006	[33]
8.	China, southern Yunnan province ***	Group A	Local rural inhabitants, random population sample	Developing economy, 3	11.7% (21/180)	Stool examination	Not reported		[17]
9.	Northern Ghana	Group A	Local inhabitants, random population sample	Developing economy, 2	11.6% (2349/20250)	Stool examination	Not reported	Study conducted from 1995 to 1998	[34]
10.	Northern Thailand	Group A,C	Local inhabitants excluding pregnant, lactating or with heart diseases	Developing economy, 3	15.9% (114/697)	Stool examination		Study conducted from April 2004 to September 2004	[35]
11.	Appalachia regions, Kentucky, US ***	Group C,D	Local inhabitants, clinic patients	Developed economy, 4	1.9% (7/378)	Serology	Not reported	All used outdoor toilet	[21]
12.	Spain, Barcelona	Group C	Immigrants from endemic areas, few locals	Developed economy, 4	17.7% (33/190) 46% (33/71)	Stool examination Serology	Gastrointestinal symptoms (64%), dermatologic symptoms (32%), neurologic symptoms (1%)	Study conducted from 2003 to 2012	[19]
13.	Cambodia	Group A	Refugees	Developing economy, 1	24.7% (40/162) 77.2% (125/162)	Stool examination Serology	Not reported	Arrived to Canada between 1982 and 1983	[36]
14.	Spain, Valencia, Gandia ***	Group C	Local farm workers, random population sample from the tourist area	Developed economy, 4	12.4 % (31/250)	Stool examination (agar plate culture)	Gastrointestinal symptoms, skin symptoms (no predominance among the infected group)	No information obtained on travelling details	[37]
15.	Africa	Group A, B, C	Sudan refugees Somali Bantu refugees	Developing economy, 1	46% (214/462) 23% (23/100)	Serology Serology	Chronic abdominal pain (not associated with the infection prevalence)	Resettled in the US in previous 5 years	[38]
16.	Jamaica	Group A	Clinical strongyloidiasis patients and controls (neighboring households)	Developing economy, 3	8.2% (17/207) 30% (62/207)	Stool examination Serology	Not reported		[39]
17.	Far East and Southeast Asia	Group A, C, D	Former WWII Far East prisoners, diagnosed with strongyloidiasis and controls	Developing economy, 2,3	12% (248/2072)	Stool examination and serology	Larva currens rash (70%)	Study conducted from 1968 to 2002, Liverpool, UK	[40]
18.	Sub-Saharan Africa, Maghreb and Latin America	Group A, B, C	Immigrants, strongyloidiasis patients	Developing economy, 2,3	90.4% (284/314) 22.9% (67/293)	Serology Stool examination	Gastrointestinal symptoms (abdominal pain, diarrhea, pruritus	Study conducted from 2004 to 2012, Southern Spain	[18]
19.	Africa, Eastern Europe, Southeast Asia, South America, the Caribbean, and the Middle East	Group A, B, C	Refugees	Developing economy, economy in transition, 1,2,3	39% (45/119)	Serology	Asymptomatic	Boston, Massachusetts	[8]
20.	Southeast Asia (Kampuchea, Laos, Vietnam	Group A,C	Immigrants, random population sample	Developing economy, 2	64.7% (125/193) 25%	Serology Stool examination	Not reported	Quebec, Canada	[41]
21.	Spain, Mediterranean coast,	Group C	Strongyloidiasis patients (ex and current farm-workers and family members), local inhabitants	Developed economy 4	0.9% (152/16607)	Stool examination (agar plate culture)	Asymptomatic (77%); Gastrointestinal symptoms (11%); cutaneous symptoms (4%); respiratory symptoms (1%); mixture of all the symptoms (7%)	Study conducted from 1990 to 1997, none travelled to the endemic areas	[42]
22.	Northeastern Thailand	Group A	Rural and urban population	Developing economy, 3	23.5% (289.8/1233)	Stool examination	Not reported	Study conducted from July to September 2002	[43]
23.	Australia, Northern territory ***	Group A	Royal Darwin Hospital patients	Developed economy, 4	33% (68/205)	Stool examination	Gastrointestinal symptoms (72%)	12 month study	[20]
24.	India, Assam	Group A,B, C	Local inhabitants, random population sample	Developing economy, 2	8.5 % (17/198)	Stool examination	Gastrointestinal, respiratory and cutaneous symptoms (29%)	Locals are mostly farm-workers	[44]
25.	Malaysia	Group A	Orang Asli community	Developing economy, 3	0% (0/54) 31.5% (17/54) 5.6% (3/54)	Stool examination Serology PCR	Not reported		[11]
26.	Palestine, Gaza Strip, Beit Lahia	Group B	Local inhabitants, random population sample, 3–18 years	N/a	5.6% (90/1600)	Stool examination	Not reported	Agricultural region	[45]
27.	Brazil, Bahia	Group A, C	AIDS Clinic patients, HIV positive and negative groups, random population sample	Developing economy, 3	1.05% (59/5608)	Stool examination	Gastrointestinal symptoms among HIV positive	Study conducted from 1997 to 1999	[46]
28.	Argentina (North) ***	Group C	Local patients at the hospital	Developing economy, 3	29.4% (67/228)	Stool examination	Not reported		[47]
29.	U.S.	Group B, C, D	Cancer treated patients	Developed economy, 4	0.25% (25/10000)	Stool examination	Fever (28%), gastrointestinal symptoms (68%), pruritic skin rash,	Cases between 1971 and 2003 22/25 are US residents	[48]
30.	Northeast Thailand	Group A	Local rural inhabitants	Developing economy, 3	28.9% (96/332) 47.5% (57/120)	Stool examination Serology	Not reported	Study conducted between October–November 2000	[49]
31.	Africa (48%), Asia (34%), Caribbean (20%), South America (3%)	Group A, B, C	Immigrants from endemic countries, travelers, Hospital for Tropical Diseases patients	Developing economy, 1,2,3	53.1% (102/192) 94.6% (157/166)	Stool examination Serology	Bowel upset, gastrointestinal symptoms, skin symptoms	Study conducted between 1991 and 2001, London	[50]
32.	Bangladesh, Dhaka	Group A, C	Local inhabitants of a slum	Developing economy, 1	23.1% (34/147) 10.2% (15/147) 61.2% (90/147)	Stool examination Stool examination (agar plate culture) Serology	Diarrhea (19%)	Study conducted from November 2009 to January 2010	[51]
33.	Nigeria, llorin	Group A	HIV clinics patients, HIV seropositive and seronegative patients	Developing economy, 2	12.2% (22/180)	Stool examination	Not reported		[52]
34.	Southeastern Brazil, Uberlandia	Group A, C	Elderly, randomly selected from nursing homes and non-institutionalised	Developing economy, 3	5% (10/200)	Stool examination	Asymptomatic		[53]
35.	Australia, Queensland, Doomadgee ***	Group B, C	Children in aboriginal communities	Developed economy, 4	27.5% (92/334)	Stool examination	Not reported	During the wet season	[23]
36.	Northern Cambodia	Group A	Local inhabitants, random population sample	Developing economy, 1	44.7% (1071/2396)	Stool examination	Not reported	Farmers (48.5%), pupils (33%)	[54]
37.	Kazakhstan ***	Group D	Adopted children, lived in orphanage	Economy in transition, 3	42.8% (3/7)	Serology	Not reported	Study in Belgium	[22]
38.	USSR, North Caucasus ***	Group D	Local inhabitants, random population sample	Developing economy, 2	0.77% (89/11530)	Stool examination	Not reported		[55]
39.	Japan, Okinawa ***	Group B	Local hospital patients	Developed economy, 4	3.4% (113/3292)	Stool examination (agar plate culture)	Not reported	*S. stercoralis* is higher in *B. hominis* infected, the last is indicator for poor hygiene	[56]

* Koppen climate classification major categories: Group A—tropical moist climate; Group B—subtropical, dry climate; Group C—subtropical, mediterranean, moist mid-latitude climates with mild winters; Group D—continental, moist mid-latitude climates with cold winters; Group E—polar climate; Group H—highland climate; ** Country’s income level categories: 1—low-income; 2—lower-middle-income; 3—upper-middle-income; 4—high-income; *** Strongyloidiasis cases in these countries are shown as a “star” sign on a map in Figure 1.
